# Tracing 600 years of long-distance Atlantic cod trade in medieval and post-medieval Oslo using stable isotopes and ancient DNA

**DOI:** 10.1098/rspb.2024.2019

**Published:** 2024-11-27

**Authors:** Lourdes Martínez-García, Angélica Pulido, Giada Ferrari, Anne Karin Hufthammer, Marianne Vedeler, Alex Hirons, Catherine Kneale, James H. Barrett, Bastiaan Star

**Affiliations:** ^1^ Department of Biosciences, Centre for Ecological and Evolutionary Synthesis, University of Oslo, Oslo NO-0371, Norway; ^2^ Department of Ecology and Evolution, University of Lausanne, Lausanne CH-1015, Switzerland; ^3^ Department of Natural History, The University Museum, University of Bergen, Bergen NO-5020, Norway; ^4^ Museum of Cultural History, University of Oslo, Oslo NO-0164, Norway; ^5^ Department of Archaeology, McDonald Institute for Archaeological Research, University of Cambridge, Cambridge CB2 3DZ, UK; ^6^ Department of Archaeology and Cultural History, NTNU University Museum, Norwegian University of Science and Technology, Trondheim NO-7012, Norway

**Keywords:** cod trade, historical fisheries, aDNA, stable isotopes, long-distance fish trade

## Abstract

Marine resources have been important for the survival and economic development of coastal human communities across northern Europe for millennia. Knowledge of the origin of such historic resources can provide key insights into fishing practices and the spatial extent of trade networks. Here, we combine ancient DNA and stable isotopes (δ^13^C, δ^15^N, non-exchangeable δ^2^H and δ^34^S) to investigate the geographical origin of archaeological cod remains in Oslo from the eleventh to seventeenth centuries CE. Our findings provide genetic evidence that Atlantic cod was obtained from different geographical populations, including a variety of distant-water populations like northern Norway and possibly Iceland. Evidence for such long-distance cod trade is already observed from the eleventh century, contrasting with archaeological and historical evidence from Britain and other areas of Continental Europe around the North and Baltic Seas, where such trade increased during the thirteenth to fourteenth centuries. The genomic assignments of specimens to different populations coincide with significantly different δ^13^C values between those same specimens, indicating that multiple Atlantic cod populations living in different environments were exploited. This research provides novel information about the exploitation timeline of specific Atlantic cod stocks and highlights the utility of combining ancient DNA (aDNA) methods and stable isotope analysis to describe the development of medieval and post-medieval marine fisheries.

## 1. Introduction

The demand for marine fish as a food source or as trading assets from growing global communities has increased in magnitude over the past millennia [1]. Understanding the extent of historical fish-trading networks is therefore key to identifying locations with long-term exploitation and ensuring their current sustainable management. In the Atlantic Ocean, marine fisheries significantly influenced the development of medieval and post-medieval European societies [1]. Long-distance fish trade from production sites in the north (e.g. northern Norway and/or Iceland) to urban centres in Britain and mainland Europe is well documented by historical and archaeological sources for medieval and early modern times, being especially evident by the thirteenth and fourteenth centuries [2–4]. Nonetheless, for the earlier Middle Ages, temporal and spatial patterns of exploitation and long-distance trade remain poorly documented. While the earliest known example of long-distance trade of Atlantic cod (*Gadus morhua*) presently has a *terminus ante quem* of *ca* 1066 CE (by which date northern Norwegian cod was brought to Haithabu in what is now Schleswig-Holstein) [5], the species was predominantly locally acquired in England and Flanders during the tenth to twelfth centuries [6–8]. Thereafter, an increasingly commercialized long-range trade of air-dried Atlantic cod (*stockfish*) only appeared from the thirteenth to fourteenth centuries onwards, around the southern North Sea and the eastern Baltic Sea [6–8]. This dried fish was likely traded via Bergen to medieval centres across Europe (Germany, Sweden, Poland, Estonia and England) [3,6,7,9–12]. Nonetheless, the development of the early medieval Atlantic cod trade remains to be discovered, between the early outlier of *ca* 1066 CE at Haithabu and the more widespread boom of the thirteenth and fourteenth centuries. A promising location to do so is within the milieu of medieval Scandinavia, where processed fish—in particular *stockfish*—was produced and found a ready cultural reception within local foodways [13]. One such location is Oslo, Norway, which emerged as a prominent town during the tenth to eleventh centuries [14,15]. Large numbers of fish remains (especially of Atlantic cod) [4] have been found during the archaeological excavation of Oslo’s urban settlement layers [16,17]. By the fourteenth century, Oslo had become an important town and centre of consumption, although not a major hub for the transshipment of processed fish [18]. By that time, participation in long-range fish trade is likely to be present. However, earlier patterns and trends of trade through time remain poorly understood.

Recent advances in biomolecular analyses, including ancient DNA (aDNA) and stable isotope approaches, have made it possible to help assign individual fish bones to specific geographical populations or geographical areas. For instance, genetic assignments can be used to detect spatiotemporal changes in the distribution of historical fish populations [19,20] or detect historical fish trade in those cases where local catches can be excluded [5,8]. The application of genetic methodology has also contributed to our understanding of fishing strategies [21,22]. Similarly, stable isotope values measured on bone collagen can also contribute to the assignment of fish to specific geographic regions as these values are influenced by the length of the food web, water temperature and salinity [6,23,24]. Considering that stable isotope values are incorporated into bone collagen predominantly via diet, they can provide information about migratory and habitat use of different species, including fish [6,25–28]. However, the differential geographical resolution of these approaches [6,8], the lack of sufficient DNA [8] or collagen preservation [29] may constrain the number of specimens that can be successfully analysed. Thus, combining these approaches in determining the provenance of economically important species like Atlantic cod can provide complementary evidence to investigate the expansion of medieval marine fisheries and the potential increase in the exploitation of targeted populations.

Here, we used such a multidisciplinary approach, combining genome-wide aDNA and stable isotopes, to describe the development of long-distance trade to Oslo over a period of approximately 600 years during the medieval and post-medieval periods, starting *ca* 1000 CE. We analysed a total of 106 archaeological specimens using low-coverage whole-genome aDNA approaches (35 out of the 106 specimens) and/or stable carbon (δ^13^C), nitrogen (δ^15^N), non-exchangeable hydrogen (δ^2^H) and sulphur (δ^34^S) isotope values (86 or 64—after quality thresholds—out of 100 specimens). We aimed to identify ecological differences between these specimens (given their isotopic values, cranial or postcranial bone elements and estimates of body size) and to determine their geographical population source based on genome-wide sequencing. Integrating both approaches, our observations support a diverse origin of Atlantic cod specimens in this assemblage, including specimens obtained through long-distance trade since *ca* 1000 CE.

## 2. Material and methods

### (a) Sample collection

**Figure 1.**
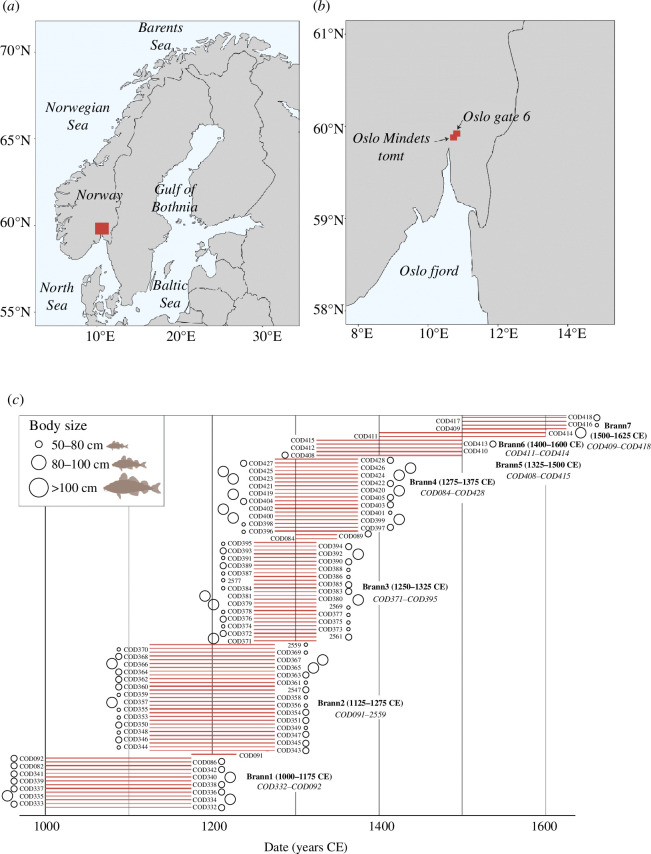
Geographical location of the archaeological Atlantic cod specimens collected from two archaeological sites in (*a*) southeast Norway and (*b*) Oslo (Oslogate 6, *n* = 100 and Oslo Mindets tomt, *n* = 6; electronic supplementary material, table S1). (*c*) Date estimation range (in red) and body size estimation (in circles) for archaeological samples. Specimens derive from layers placed between distinct fire layers (*Branntrinn*) that have been dated using archaeological methods (across the eleventh to seventeenth centuries). A total of eight fire stages (Brann1 to Brann8) have been identified and are used to describe the stratigraphy and constructions in urban development [17]. Specimens COD406 and COD407 (from Brann8) do not have a confident date estimation and are excluded from this figure. Body size is significantly different between fire layers, with fish from Brann4 significantly larger than those from Brann2 and Brann3.

We have analysed 106 Atlantic cod bone samples collected from two archaeological sites: Oslogate 6 (*n* = 100) and Oslo Mindets tomt (*n* = 6; electronic supplementary material, table S1). The zooarchaeological assemblages (bones) from both sites are stored in the osteological collections at the University Museum, University of Bergen, under the museum numbers JS-784 (Oslogate 6) and 537 (Oslo Mindets tomt). Oslogate 6 (59.91°N, 10.77°E) was located in the northern part of medieval Oslo [17]. This site was excavated during 1987–1989, while Oslo Mindets tomt (59.90°N, 10.76°E) was excavated in 1973 (figure 1). Remains of barley, fish bones and animal dung (likely from livestock) have been found in the area, while increases in large leather deposits by the late twelfth century in Oslogate 6 are indicative of the development of trading activities (i.e. shoemaking) in the town [17,30].

Samples—cranial and postcranial elements—have been stored dry and unfrozen after field collection. All specimens were morphologically (see Archive: Osteological collections, University Museum, University of Bergen) and genetically identified as Atlantic cod. Samples have been dated based on the archaeological context (e.g. fire layer) including carbon-14 dating, dendrochronology and typology (for more information, see [17]). Specifically, samples at Oslogate 6 are distributed between seven fire stages (Brann1-7) dated using archaeological methods (across the eleventh to seventeenth centuries) and are used to describe the stratigraphy and constructions in urban development (i.e. waste layers) (for details about the fire layers, see [17]). An eighth layer (Brann8) has been described for Oslogate 6, which includes an uppermost layer that is associated with modern times (i.e. archaeological remains younger than 1624 CE [17]). Samples across the fire layers at Oslogate 6 are distributed as follows (electronic supplementary material, table S1): Brann1 (oldest, where 12 out of 14 samples were processed for genomic analysis), Brann2 (*n* = 7/30), Brann3 (*n* = 8/27), Brann4 (*n* = 10/22), Brann5 (*n* = 5/5), Brann6 (*n* = 2/2), Brann7 (*n* = 4/4) and Brann8 (*n* = 2/2). Oslo Mindets tomt samples are dated by the archaeological context [31] and are analysed together with those specimens from Oslogate 6 with which they co-occur in time [32]. Three specimens (COD082, COD086 and COD092) co-occur in time with Brann1, one specimen (COD091) co-occurs with Brann2 and two specimens (COD084, COD089) co-occur with Brann4.

### (b) aDNA extraction, library preparation and sequencing

A total of 50 samples were processed in the aDNA laboratory at the University of Oslo [33,34] (electronic supplementary material, tables S1 and S2). Samples were treated as per Ferrari *et al*. [35] and Martínez-García *et al.* [36] before DNA extraction. Genomic DNA was extracted using a mild bleach treatment and pre-digestion step protocol [37]. Double-indexed blunt-end sequencing libraries were built using either the Meyer–Kircher protocol [38,39] with modifications by Schroeder *et al*. [40] or the single-stranded Santa Cruz reaction protocol (tier 4) ([41]; electronic supplementary material, table S1). Library quality and concentration were examined with a High Sensitivity NGS Fragment Analysis Kit on the Fragment Analyzer^TM^ (Advanced Analytical). Libraries were sequenced on the Illumina HiSeq 4000 or on the Novaseq 6000 platform at the Norwegian Sequencing Centre (electronic supplementary material, table S1). Sequencing reads were processed using PALEOMIX v. 1.2.13 [42] and AdapterRemoval v. 2.1.7 [43] to trim residual adapter contamination, to collapse overlapping paired reads, and to filter reads with excessive missing nucleotides. Filtered reads were aligned using the gadMor2 genome as reference [44,45] using BWA v. 0.7.12 [46] with the *backtrack* algorithm, disabled seeding and minimum quality score of 25. aDNA deamination patterns were characterized using mapDamage v. 2.0.9 [47].

### (c) Genomic analysis

**Figure 2.**
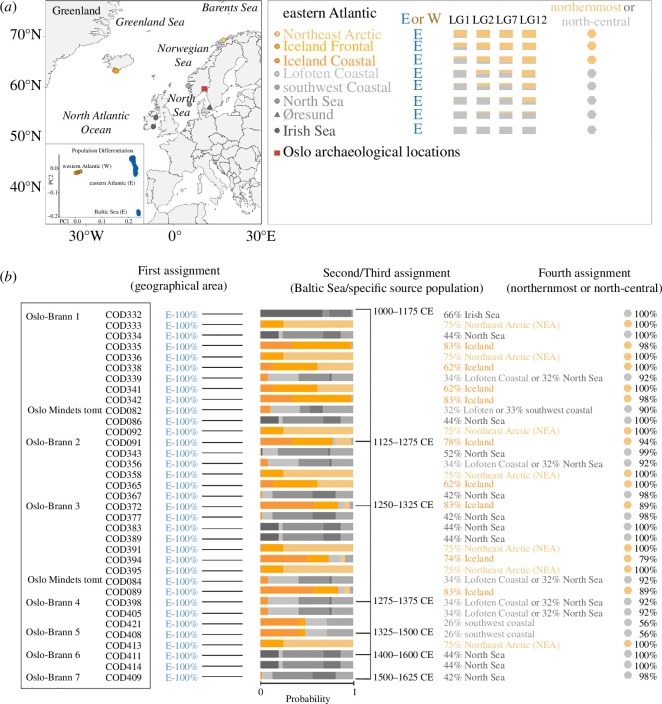
Genetic analyses of the archaeological Atlantic cod specimens from Oslo. (*a*) Geographical distribution of modern inversion frequencies of chromosomal inversions in Atlantic cod (LG1, LG2, LG7 and LG12) from reference populations across the North Atlantic Ocean. The map is modified from Martínez-García *et al*. [8]. Alleles associated with a northernmost (Northeast Arctic and Iceland) composite genotype distribution are assigned in orange. Alleles associated with a north-central (Norwegian Coast, the North Sea, the Irish Sea, Øresund) genotype distribution are assigned in grey. Oslo’s archaeological sites are indicated with a red square. (*b*) Genomic assignments to a geographical area, a source population and a genotypic group are based on the frequencies of chromosomal inversions of Atlantic cod (LG1, LG2, LG7 and LG12) as per Star *et al*. [5]. Percentages (%) indicate the highest probability of being from one population and area.

To determine the biological origin of 50 Atlantic cod specimens, we followed the hierarchical approach described in Martínez-García *et al*. [8]. First, we used the genome-wide approach in the BAMscorer pipeline [48] to assign ancient cod specimens to the eastern or western Atlantic Ocean. Second, among those specimens with an eastern Atlantic origin, we used a similar approach to identify any specimen with a Baltic Sea origin. Third, we used the chromosomal inversion approach to determine the individual haplotypes of the four major chromosomal inversions in Atlantic cod (LG1, LG2, LG7 and LG12) [49] associated with migratory behaviour and temperature clines [49–53]. These combined genotype distributions can indicate an affinity towards a particular ecotype [5,8]. We included reference data on (modern) inversion frequencies from the following populations: the Northeast Arctic (NEA), Iceland (frontal and coastal ecotypes, which differ in their tendency for long-distance migration), the Norwegian Coast (Lofoten and southwest), the North Sea, the Irish Sea and Øresund (figure 2*a*) [5,53,54]. Samples with >0.1% endogenous DNA and an average of >50 single nucleotide polymorphisms across all four chromosomal inversions have been included for specific genomic assignments (electronic supplementary material, table S1). For each individual specimen, the source population with the highest percentage was considered the likely geographical population source (electronic supplementary material, tables S2 and S3). Specimens with equal or similar assignment probabilities for two populations have both populations as their putative origin (electronic supplementary material, tables S2 and S3). Finally, we recognized two spatially larger scale distinct groups (northernmost and north-central) as described in Martínez-García *et al*. [8]. The northernmost group includes NEA and Iceland (adding probabilities of both Icelandic ecotypes), while the north-central group includes the Norwegian Coast (coastal Atlantic cod from Lofoten and southwest Norway), the North Sea, the Irish Sea and Øresund [8]. While our ability to obtain high affinities of ancient specimens to specific populations within these two distinct regions is often low, we consider those that fall with high probability within the northernmost group (see §3, figure 1*b*) as specimens that must have been obtained through long-distance trade following Martínez-García *et al*. [8]. In contrast, we cannot exclude a putatively local geographical population source (i.e. near Oslo) for those specimens belonging to the north-central group.

We investigated possible associations between the origin of 27 specimens assigned with a >70% probability to a northernmost or north-central group (electronic supplementary material, table S2) and their fish bone element (cranial (premaxilla, articular, dentary, maxilla) or postcranial (vertebra and cleithrum)) [8] using the Fisher’s exact test. Full details of the criteria used to exclude samples for statistical analyses are provided in the electronic supplementary material (table S2).

Additionally, we used the Fisher’s exact test to evaluate whether the binary genetic assignment to the north-central or northernmost groups changed over time (>70% probability). We also evaluated whether the probability—scaled from 0 to 1—of having an NEA or Icelandic origin (adding probabilities of both Icelandic ecotypes) differs over time (i.e. fire layers), body size or bone element. Here, we used logistically transformed probabilities (*logit*) of NEA or Icelandic origin after testing for normality with a Shapiro–Wilk test. Thereafter, we performed non-parametric Kruskal–Wallis tests between such variables. Only fire layers with sufficient sample sizes (*n* ≥ 4 in Brann1 (1000–1175 CE) to Brann4 (1275–1375 CE)) were used in these analyses (*n* = 29). Finally, it is possible that fish coming from distinct geographical population sources may originally have differed in size, or that different fishing practices may themselves select for a particular size range in their catches. Given their often-fragmented condition, archaeological bones were assigned to total (length) body-size categories based on comparison with 1 : 1 scale scans of reference specimens from the known size of Atlantic cod. Using the Fisher’s exact test, we further investigated whether there were significant differences between the estimated total (length) size categories (50–80, 80–100 and >100 cm) in specimens that were genetically assigned to the north-central or northernmost group, or to northern Norway or Iceland.

### (d) Stable isotope analysis

Stable isotope analysis was performed on 100 Atlantic cod specimens (50 of which were also processed for genomic sequencing as noted above; electronic supplementary material, table S1). We measured stable carbon (δ^13^C), nitrogen (δ^15^N), non-exchangeable hydrogen (hereafter described as δ^2^H for simplicity, which represents *in vivo* values rather than the exchange with atmospheric water vapour [28]) and sulphur (δ^34^S) on purified bone collagen. We processed a cross section of the bone material—for an approximate lifetime average for the isotopic values and thus reducing the impact of changing trophic level associated with age [55]—following the protocols of Barrett *et al*. [56] and references therein, with the exclusion of a lipid removal step. For δ^13^C values and δ^15^N values, the extracted collagen was analysed in triplicate at the University of Cambridge using a Costech elemental analyser coupled to a Thermo Finnigan Delta V Isotope Ratio Mass Spectrometer (IRMS). δ^2^H and δ^34^S values (measured by Iso-Analytical Limited, by elemental analyser IRMS) were analysed routinely in duplicate, although some samples yielded only enough collagen for single measurements. All isotope data are reported using international scales: δ^13^C values are reported relative to Vienna Peedee Belemnite (VPDB), δ^15^N values to AIR, δ^2^H values to Vienna Standard Mean Ocean Water (VSMOW) and δ^34^S to Vienna-Cañon Diablo Troilite (VCDT). The reported non-exchangeable δ^2^H values are corrected for exchangeable hydrogen by three-point linear calibration using standards. Overall, 64 samples passed appropriate quality-control thresholds for all four measured isotope values (atomic C/N ratio of 2.9–3.6, atomic C/S ratio of 125–225, atomic N/S ratio of 40–80) [57]. An additional 22 samples only passed appropriate C/N ratio quality-control thresholds. Therefore, the δ^13^C, δ^15^N and δ^2^H values are probably reliable for these 22 specimens, but potentially not the δ^34^S values. For this reason, analyses using only δ^13^C, δ^15^N and/or δ^2^H values (individually and interacting) are based on 86 specimens, while analyses including δ^34^S values (individually and in combination with other data) are based on 64 specimens.

To evaluate the impact of *post-mortem* processes on the isotopic values we investigated correlations between C : N ratios and δ^13^C values. To investigate potential interactions between isotopes, we evaluated the normality of our data with a Shapiro–Wilk test, followed by correlations between δ^13^C–δ^15^N, δ^15^N–δ^2^H and δ^13^C–δ^34^S values. We implemented a Spearman’s correlation between C : N ratios and δ^13^C values (*n* = 86), and between δ^13^C and δ^34^S values (*n* = 64), while Pearson’s correlations were used between δ^13^C and δ^15^N values (*n* = 86) and δ^15^N and δ^2^H values (*n* = 86). Based on age, growth and environmental and ecological shifts over time, we investigated the variability of isotope values over time and across body size groups. We performed ANOVA analysis for δ^13^C, δ^15^N (*n* = 86) and δ^2^H values (*n* = 83, excluding individuals with missing δ^2^H values) followed by a Tukey HSD *post hoc* test; while a non-parametric Kruskal–Wallis test was computed for δ^34^S values (*n* = 64) followed by a Dunn *post hoc* test with a Bonferroni correction (<3 groups).

To identify potentially different trophic levels and/or exposure to ecological conditions among specimens, we examined the distribution of isotope values (δ^13^C, δ^15^N and δ^2^H) within each of the three body size categories. We further evaluated the differences between the body sizes of all archaeological specimens distributed across Brann1 to Brann4 (*n* = 86) using a Kruskal–Wallis test, followed by a *post hoc* Dunn test with a Holm correction (>3 groups).

### (e) Combined genome–isotope analyses

A summary of isotope values, in relation to all body size categories and the putatively genomic assignment of individuals to either a NEA or Iceland or a north-central or northernmost origin, was represented by a principal component analysis (PCA) on the 64 relevant δ^13^C, δ^15^N, δ^2^H and δ^34^S individual values. We performed an ANOVA analysis to describe the contribution of size and time (Brann1 to Brann4) to each principal component (PC) in the PCA followed by a Tukey HSD *post hoc* test.

To investigate whether the isotopic values differ when individuals are genomically assigned to either NEA or Iceland (adding the probabilities of both Icelandic ecotypes), we computed a multivariate linear regression with logistically (*logit*) transformed probabilities (scaled from 0 to 1) of NEA or Icelandic origin (*n* = 20) as dependent variables and δ^13^C, δ^15^N, δ^2^H and δ^34^S values as predictors. We tested the regression for homoscedasticity with a Breusch–Pagan test and validated the model by assessing residuals and symmetry. We further evaluated for multicollinearity by removing δ^15^N from our regressions (after significant relationships with other isotope values; see §3). We did not observe major differences when removing δ^15^N; therefore, the final regression model includes all four isotope values. Additionally, considering that *stockfish* is primarily produced from the Atlantic cod migratory ecotype in Norway, we selected those specimens genomically assigned to the Iceland frontal migratory ecotype (another source of *stockfish* production) to assess the differences in isotopic values between migratory ecotypes. Multivariate linear regressions with specimens with an Icelandic frontal affinity follow the same procedure previously explained.

Finally, we assessed the direct relation between a binary assignment to NEA or Iceland (adding both Icelandic ecotypes) and the δ^13^C, δ^15^N, δ^2^H and δ^34^S values. We used one-way ANOVAs (for δ^13^C, δ^15^N and δ^34^S) or Welch’s ANOVAs (for δ^2^H) after testing for equal variances with a Bartlett test. Only specimens with a >70% probability NEA or overall >70% probability Icelandic assignment were included (electronic supplementary material, table S2). Therefore, 10 specimens were tested against δ^13^C, δ^15^N and δ^2^H values and nine specimens were tested against δ^34^S values. Icelandic frontal (migratory ecotype) assignments are usually shared with an Icelandic coastal (stationary ecotype) assignment (electronic supplementary material, table S3). Consequently, an Icelandic frontal assignment alone cannot be binary assigned with >70% probability of origin to a population. Thus, we only included samples with a confident (>70% probability) overall Icelandic origin. All statistical analyses have been performed in R [58]. Full details of the packages are provided in the electronic supplementary material.

## 3. Results

### (a) Genomic analysis

We successfully sequenced 35 out of the initial 50 Atlantic cod specimens with a total of *ca* 770 million paired reads, of which approximately 111 million reads aligned with a range of 0.1% to 47% of endogenous DNA content per specimen (electronic supplementary material, table S2). As expected, patterns of DNA fragmentation and deamination rates are consistent with those of authentic aDNA (electronic supplementary material, figure S1). We found that all 35 specimens can be assigned to an eastern Atlantic origin (100% assignment probability; figure 2*b* and electronic supplementary material, tables S1 and S2), of which 19 specimens had the highest affinity to the north-central group (56–100% assignment probability). No specimens were identified with a Baltic Sea origin. Within the north-central group, specific population assignments are statistically uncertain, with 10 specimens putatively assigned to the North Sea (42–52% assignment probability), one specimen to the Irish Sea (66% assignment probability), five specimens to both the North Sea (32% assignment probability) and the Norwegian Coast (Lofoten, 34% assignment probability), one specimen to the Norwegian Coast (Lofoten and southwest, 32 and 33% assignment probability respectively) and two specimens to the southwest coast of Norway (26% assignment probability; figure 2*b* and electronic supplementary material, table S3). Sixteen specimens had the highest affinity to the northernmost group (79–100% assignment probability), of which seven specimens were assigned to the Northeast Arctic (75% assignment probability) and nine were likely assigned to Iceland, with 62–83% assignment probability (adding the probabilities of both migratory and stationary ecotypes; figure 2*b* and electronic supplementary material, table S3).

While postcranial bones have been associated with long-distance sources (i.e. NEA and Iceland) [8], we did not find a statistically significant association between the bone element (cranial or postcranial) and specimens with a local (north-central group) or traded origin (northernmost group; *p*‐value = 0.37; *n* = 27; electronic supplementary material, figure S2a, table S2). In addition, we did not find statistically significant differences over time between specimens genetically assigned to either a north-central or northernmost group (*p*‐value = 0.77; *n* = 29; electronic supplementary material, figure S2b, table S1). We did not find any statistical difference between the probability of having an NEA or Icelandic origin in bone element (NEA: Kruskal–Wallis χ^2^ = 2.28, d.f. = 1, *p*‐value = 0.13 and Iceland: Kruskal–Wallis *χ*^2^ = 0.13, d.f. = 1, *p*‐value = 0.72; *n* = 33; electronic supplementary material, figure S3a,b), in body size (NEA: Kruskal–Wallis *χ*^2^ = 2.00, d.f. = 2, *p*‐value = 0.37 and Iceland: Kruskal–Wallis *χ*^2^ = 0.25, d.f. = 2, *p*‐value = 0.88; electronic supplementary material, figure S3c,d) or across time (Brann1 to Brann4; NEA: Kruskal–Wallis *χ*^2^ = 0.74, d.f. = 3, *p*‐value = 0.86 and Iceland: Kruskal–Wallis *χ*^2^ = 0.04, d.f. = 3, *p*‐value = 0.99; electronic supplementary material, figure S3e,f). Furthermore, we did not find statistical differences between the size categories of specimens genetically assigned to Iceland and NEA (*p* = 0.09, *n* = 16), the north-central group and Iceland (*p* = 0.68, *n* = 25), the north-central group and NEA (*p* = 0.46, *n* = 23), the north-central group, NEA and Iceland (*p* = 0.26, *n* = 32) and the north-central group and northernmost group (*p* = 1.0, *n* = 32; electronic supplementary material, table S4).

### (b) Isotope analysis

We successfully extracted collagen from 93 out of 100 Atlantic cod specimens from Oslogate 6, with 86 passing quality thresholds for δ^13^C, δ^15^N and δ^2^H values (electronic supplementary material, table S1). For δ^34^S values, 64 specimens passed quality control thresholds. Collagen yields of all 93 extractions ranged from 1.5 to 10.6%. Isotope values that passed initial quality control thresholds ranged from −15.9 to −11.9‰ (mean −13.9‰, variance 0.8, s.d. = 0.9) for carbon (δ^13^C), from +12.7 to +16.8‰ (mean +14.9‰, variance 0.8, s.d. = 0.9) for nitrogen (δ^15^N), from −13.8 to +38.1‰ (mean +12.9‰, variance 116.9, s.d. = 10.8) for hydrogen (δ^2^H) and from +6.0 to +17.5‰ (mean +14.0‰, variance 5.3, s.d. = 2.3) for sulphur (δ^34^S; electronic supplementary material, table S5).

The C : N ratios for the included data ranged from 3.0 to 3.6 (electronic supplementary material, figure S4, table S1) and did not have a significant correlation with δ^13^C values (Spearman’s *ρ* = −0.19, *p*‐value = 0.07). Therefore, we assume that *post-mortem* processes did not significantly impact our isotopic data [29]. Isotope values were strongly correlated, with a significant relation between δ^15^N and δ^13^C values (Pearson’s correlation = 0.46, *p*‐value ≤0.01; electronic supplementary material, figure S5a) and between δ^2^H and δ^15^N values (Pearson’s correlation = 0.51, *p*‐value ≤0.01; electronic supplementary material, figure S5b). No significant relationship was observed between δ^13^C and δ^34^S values (Spearman’s correlation *ρ* = 0.18, *p*‐value = 0.15; electronic supplementary material, figure S5c).

Body size significantly influenced three isotope values (electronic supplementary material, table S6a): δ^13^C (ANOVA: *F-*value = 6.70, *p*‐value ≤ 0.01, *n* = 86; electronic supplementary material, figure S6a), δ^15^N (ANOVA: *F-*value *=* 5.47*, p‐*value ≤ 0.01, *n* = 86; electronic supplementary material, figure S6b) and δ^2^H (ANOVA: *F-*value = 7.38*, p‐*value ≤ 0.01, *n* = 83; electronic supplementary material, figure S6c). Such significant differences in δ^13^C, δ^15^N and δ^2^H values were found between the smallest (50–80 cm) compared to the largest (>100 cm) body size categories (Tukey HSD *post hoc* test *p‐*value ≤ 0.01; electronic supplementary material, table S6a). Interestingly, we observed a bimodal distribution within medium-sized fish (80–100 cm) for δ^13^C and δ^15^N values but not in δ^2^H values or smaller and larger body size categories (electronic supplementary material, figure S7). Furthermore, we found significant differences of δ^13^C (ANOVA: *F-*value = 5.91, *p*‐value ≤ 0.01, *n* = 81; electronic supplementary material, figure S8a), δ^15^N (ANOVA: *F-*value = 3.94, *p*‐value ≤ 0.01, *n* = 81; electronic supplementary material, figure S8b) and δ^2^H values (ANOVA: *F-*value = 3.67, *p*‐value ≤ 0.01, *n* = 79; electronic supplementary material, figure S8c) across time (Brann1 to Brann4; electronic supplementary material, table S6b). Specifically, Brann4 (1275–1375 CE) had significantly higher isotope values (δ^13^C *p*‐value ≤ 0.01; δ^15^N *p*‐value = 0.03 and δ^2^H *p*‐value = 0.01) compared to Brann2 (1125–1275 CE). Brann4 also had significantly higher δ^13^C values (*p*‐value ≤ 0.01) compared to Brann3 (1250–1325 CE) and significantly higher δ^15^N values (*p*‐value = 0.01) compared to Brann1 (1000–1175 CE). No significant correlations were obtained between δ^34^S values and body size (Kruskal–Wallis *χ*^2^ = 0.11, d.f. = 2, *p*‐value = 0.95, *n* = 64; electronic supplementary material, figure S6d) or across time (Kruskal–Wallis *χ*^2^ = 4.69, d.f. = 3, *p*‐value = 0.20, *n* = 60; electronic supplementary material, figure S8d). We found significant differences among the body size of our archaeological specimens across time (Kruskal–Wallis *χ*^2^ = 11.93, d.f. = 3, *p*‐value ≤ 0.01). These differences can be observed between larger fish from Brann4 against Brann2 (TukeyHSD *post hoc* test *p‐*value = 0.04) and Brann3 (Tukey HSD *post hoc* test *p‐*value = 0.04).

### (c) Combined genome–isotope analyses

**Figure 3.**
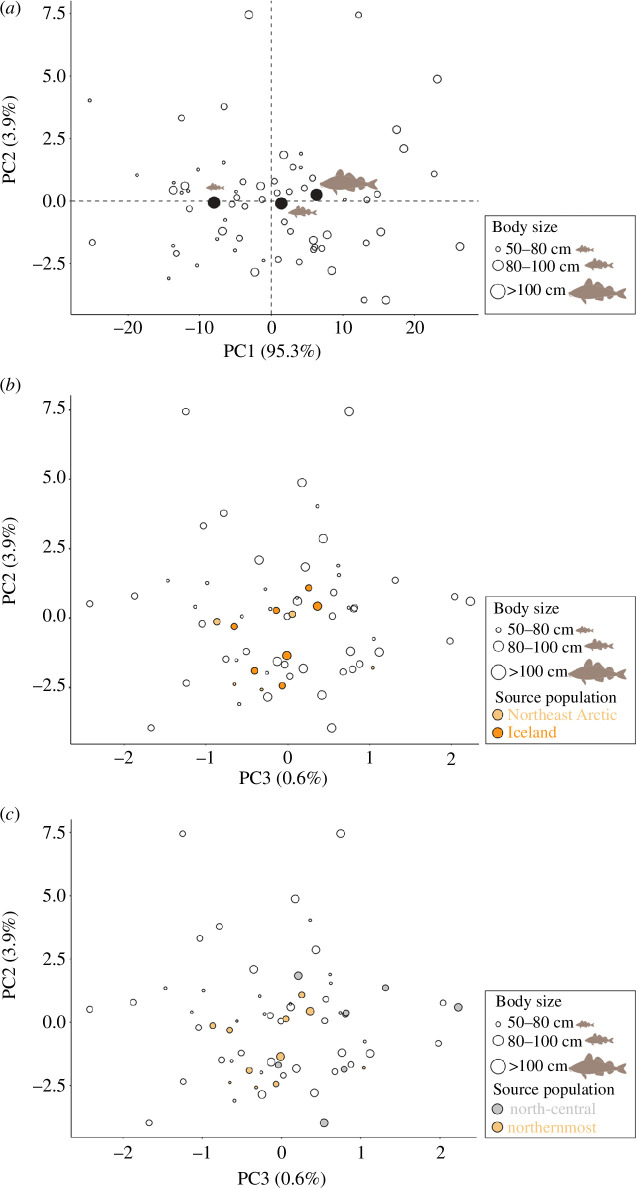
Principal component analysis (PCA) for carbon (δ^13^C), nitrogen (δ^15^N), non-exchangeable hydrogen (δ^2^H) and sulphur (δ^34^S) isotope values, highlighting (*a*) body size and (*b*) putative genetically inferred source population assignments of individuals to the Northeast Arctic (NEA) or Iceland populations and (*c*) north-central or northernmost genomic groups. Values distributed across the PC1–PC2 axes explain 99.18% of the observed variations, while values across the PC2–PC3 axes explain 4.5% of the observed variation. The means (black dots) of each particular body size class (grey cod silhouette) are sorted smallest to largest along PC1s.

PCA was employed to investigate the relationship between isotope data and genomic assignments while assessing for the contribution of body size. The first two principal component axes (PC1 and PC2) explained 99.18% of the observed variation (figure 3*a*). PC1 values significantly increased with body size (ANOVA: *F-*value = 8.85, *p*‐value ≤ 0.01, *n* = 64, figure 3*a*). Such significant differences were found between the smaller (50–80 cm) compared to the medium (80–100 cm; Tukey HSD *post hoc* test *p‐*value ≤ 0.01) and larger (>100 cm; Tukey HSD *post hoc* test *p‐*value =<0.01) body size categories. We found that the highest contribution to the observed variation in PC1 was that of δ^2^H values (PC1 contribution = 99.53%), despite similarly strong correlations with body size and δ^13^C and δ^15^N values (figure 3*a*, electronic supplementary material, table S5a,b). Importantly, no body size changes were observed across PC2 (ANOVA: *F-*value = 0.12, *p*‐value = 0.89, *n* = 64, figure 3). PC2 presented the highest contribution and a negative relation with δ^34^S values according to its loading values (PC2 contribution = 98%, electronic supplementary material, table S5a,b). Furthermore, values in PC3 increased with δ^15^N and δ^13^C values (electronic supplementary material, table S5a,b), however, no particular body size changes were observed across PC3 (ANOVA: *F*-value = 2.25, *p*‐value = 0.11, *n* = 64). In the PCAs of isotope values highlighting the genomic assignment of individuals, the second and third principal component axes (PC2 and PC3) explained 4.5% of the observed variation (figure 3*b,c*). The north-central versus northernmost genomic groups are partially separated with a noticeable overlap between regions on PC3, whereas NEA versus Iceland do not separate on PC2 or PC3 (figure 3*b,c*).

**Figure 4.**
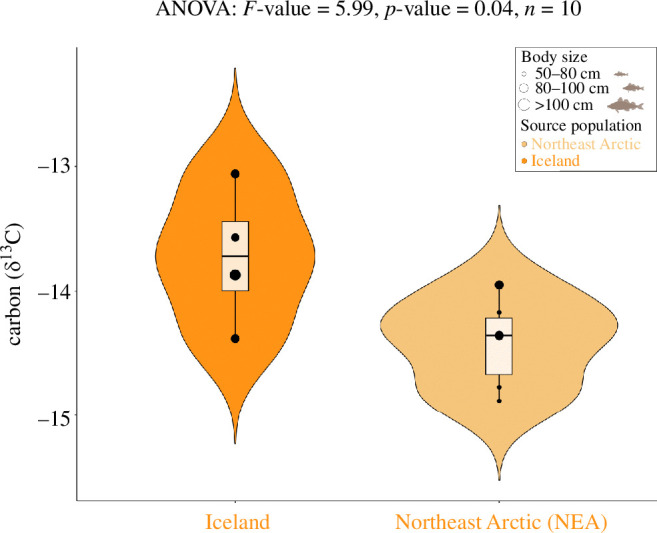
Differences in carbon (δ^13^C) values across specimens with a binary assignment to genetically inferred source populations: Iceland or NEA. Only specimens with a 70% or higher probability of being assigned to either origin were used in this analysis (see, electronic supplementary material, tables S2, S3).

Multivariate linear regressions showed a significant relationship between δ^13^C values and the (genomic) probability of having a northern Norway origin (NEA, *p*‐value = 0.01, adjusted *R*^2^ = 0.50; electronic supplementary material, table S7a), but not for the (genomic) probability of having an Icelandic (*p*‐value = 0.36; electronic supplementary material, table S7b) or Icelandic frontal origin (*p*‐value = 0.20; electronic supplementary material, table S7c). We found a significant difference between the δ^13^C values depending on the binary population assignment between an NEA or Icelandic biological origin (ANOVA: *F-*value = 5.99, *p*‐value = 0.04, figure 4). Such differences were not observed for δ^15^N, δ^2^H and δ^34^S values (electronic supplementary material, figure S9a–c). Other isotope values did not show any significant influence on the biological origin of Atlantic cod specimens (electronic supplementary material, table S7).

## 4. Discussion

We have identified different geographical population sources and ecological differences for ancient Atlantic cod bones obtained from an archaeological assemblage from medieval Oslo over a period of 600 years, by implementing a multidisciplinary approach using aDNA and stable isotopes. We have found an overall diverse provenance of Atlantic cod specimens among the eleventh to seventeenth centuries in Oslo. Atlantic cod obtained through trade (e.g. either the NEA or possibly Iceland) are present throughout time and are found in the earliest deposits. Using multivariate linear regression, we found a significant relationship between carbon (δ^13^C) values and the (genomic) probability of being assigned to NEA as the original source. Moreover, by assessing for the contribution of Atlantic cod size, PCA analysis supports a separation between the north-central and northernmost genomic groups based on stable isotope values. Our ANOVA results suggest that δ^13^C values are also significantly different between those specimens with different chromosomal inversion LG1 genotypes (NEA and Iceland). Considering that this inversion is associated with different behaviour patterns and habitat preferences in Atlantic cod [49–53], our observation suggests a diverse geographical population source of Atlantic cod specimens in medieval Oslo. These observations are consistent with known isoscape patterns in the δ^13^C values of collagen from archaeological Atlantic cod bones, with specimens from Arctic Norway having on average more negative ratios than both Icelandic specimens and Norwegian Sea/North Sea specimens from lower latitudes [6,59]. Below we describe the implications of these findings.

Previous zooarchaeological, isotopic and genetic evidence indicates an increase in long-distance trading of Atlantic cod specimens obtained from remote northern fisheries (e.g. NEA or possibly Iceland) to other locations in Europe, such as England, Flanders, Poland and Estonia, from the thirteenth to fourteenth centuries onwards [6–8,60]. Our observations show a consistent long-distance trading of Atlantic cod specimens, likely obtained from the Lofoten or Vesterålen archipelagos, since the eleventh century (*ca* 1000 CE). Notably, the *stockfish* transport from northern Norway to Haithabu—now in northern Germany—has previously been described during the eleventh century (i.e. before *ca* 1066 CE [5]). Considering that Oslo experienced a pronounced royal and ecclesiastical presence during the eleventh century [14,15,61], elite and ecclesiastical networks may have facilitated access to trading assets (e.g. food) from northern Norway. Nonetheless, Oslo was not a major hub of *stockfish* trade; therefore, it can be inferred that the Atlantic cod of northern origin present since the eleventh century were primarily for local consumption.

Following Martínez-García *et al*. [8], we genetically identified a presumed Icelandic origin of processed fish, possibly *stockfish* [8,62], with specimens assigned to Icelandic frontal (migratory behaviour) or coastal (stationary behaviour) ecotypes. The genomic differences between Icelandic and NEA cod can be found in the chromosomal inversion LG1, where a higher frequency of north-central genotypes can be found in Iceland [53]. However, there are similarities between inversion frequencies (for LG1) between deep water Iceland (migratory ecotype) and NEA cod [8,63]. Considering such similarities, and possible biological complexity, the genetic assignments in this study based on inversion frequencies to either Iceland (either frontal and coastal ecotype) or NEA remain uncertain. Nonetheless, NEA cod has distinct migratory behaviour by feeding in the Barents Sea before spawning along the Norwegian coast [64], while the Icelandic populations consist of a combination of two ecotypes with different spawning, migratory and feeding behaviours [65]. Moreover, modern otolith increment growth indicates that Icelandic cod grow faster than NEA cod during their early stages of life (up to 6 years), whereas NEA cod appears to grow faster during older years [66]. Consequently, previous studies have associated significantly higher δ^13^C values in Icelandic cod otoliths (δ^13^C_oto_) in comparison to NEA cod, with differences in fish growth and also metabolism [66]. The significant differences that we found in δ^13^C values for specimens assigned to NEA (based on multivariate linear regression) and among specimens assigned to NEA and Iceland are, therefore, consistent with these biological and ecological differences. Our observations then suggest that these specimens may have been exposed to different oceanographic and ecological conditions [67,68] and/or different diet compositions [6,64].

No overall differences in body size categories between genomic assignments to Iceland and NEA specimens, or northernmost and north-central genomic groups, were observed; hence the isotopic differentiation between these genetic assignments is probably not driven by these fish feeding at different trophic levels. In fact, considering the significant differences between body size categories across isotopic values (δ^13^C, δ^15^N and δ^2^H) and across time (fish from Brann4 (1275–1375 CE) are larger compared with those from Brann2 (1125–1275 CE) and Brann3 (1250–1325 CE)), our results reflect the expected ecological complexity during an individual’s lifetime (related to size-specific metabolic rates that decrease as fish grow older), sexual maturation (which differs according to geographical latitude) and diet composition (from lower or higher trophic levels) of different ecotypes or individuals [69–71]. Interestingly, we observed bimodal distributions of δ^13^C and δ^15^N values within the medium-size specimens (80–100 cm), which are presumed to feed at similar trophic levels given they are classified in the same body size category. These differences might either reflect differences in their environment (perhaps different locations) or distinct feeding strategies amongst individuals of different sizes [72–74]. Overall, our findings, including variability within the stable isotope data, suggest that multiple localities and/or ecotypes provided *stockfish* to Oslo from the eleventh century onwards.

## 5. Conclusion

For millennia, people have relied on Atlantic cod as a food source and key income product for developing coastal communities across northern Europe. Here, we identified a continuous occurrence of Atlantic cod obtained from remote geographical population sources like northern Norway or possibly Iceland since the eleventh century in medieval and post-medieval Oslo. Our observations on the extent of the long-distance fish trade can provide valuable information about the exploitation timeline of specific Atlantic cod stocks and provide a rationale for the long-term baseline assessment for the impacts of historic exploitation. While the interpretation of the genomic assignment of ancient fish specimens to Iceland remains uncertain based upon inversion frequencies only, the association of genetic data with differences in isotopic values does provide evidence for the existence of mixed fisheries, targeting either fish at different spatial locations or co-occurring ecotypes that supported the long-distance trade to Oslo. This study highlights the utility of combining ancient DNA methods with isotope analysis to provide complementary insights into geographical and ecological differences among zooarchaeological fish remains to describe the long-term exploitation of economically important marine species during the medieval and post-medieval development of coastal communities.

## Data Availability

The raw reads for the ancient specimens are released under the ENA accession numbers PRJEB37681 and PRJEB71940. This paper is available as a pre-print in BioRxiv [75]. Code is available on Dryad [76]. Full detail materials and methods are provided online in the electronic supplementary material [77].
